# The effect of exercise intervention on prosocial behavior in junior high school students: the chain mediation effect of empathy and interpersonal relationships

**DOI:** 10.3389/fpsyg.2025.1723840

**Published:** 2025-12-19

**Authors:** Shaohua Tang, Tianci Lu, Hanwen Chen, Baole Tao, Jun Yan

**Affiliations:** College of Physical Education, Yangzhou University, Yangzhou City, Jiangsu Province, China

**Keywords:** empathy, exercise intervention, interpersonal relationships, junior high school students, prosocial behavior

## Abstract

**Introduction:**

This study aimed to explore the effects of exercise intervention on prosocial behavior in junior high school students and its underlying mechanisms.

**Methods:**

Using convenience cluster sampling, 90 students were selected from two first-grade classes at a secondary school, with 45 assigned to the experimental group and 45 to the control group. The experimental group participated in a 12-week basketball exercise program, consisting of three sessions per week, each accumulating 30 min of moderate-to-vigorous physical activity. The control group attended regular physical education classes as scheduled in the curriculum. Psychological scales were administered to assess variable levels before and after the intervention.

**Results:**

After the intervention, significant main effects of time and interaction effects between time and group were observed in empathy, interpersonal relationships, and prosocial behavior. A significant main effect of group was also found in prosocial behavior. Moreover, empathy and interpersonal relationships mediated the relationship between exercise intervention and prosocial behavior.

**Conclusions:**

Exercise intervention can improve empathy, interpersonal relationships, and prosocial behavior among junior high school students. Specifically, basketball exercise yielded significantly greater benefits than conventional physical education. Exercise indirectly promotes prosocial behavior through the separate mediating roles of empathy and interpersonal relationships, which also form a sequential mediation pathway between exercise intervention and prosocial behavior.

## 1 Introduction

Against the backdrop of technological advancement and accelerated lifestyles, adolescents‘values and ethical conduct face profound challenges, with instances of moral misconduct and emotional detachment occurring with increasing frequency. Among these, antisocial behaviors frequently occurring during adolescence may escalate into criminal activities if left unaddressed, posing a serious threat to both individuals and society (Mazza et al., 2025). As its counterpart, fostering prosocial behavior is crucial for enhancing the overall social landscape and promoting sustained, healthy development (He et al., 2020). Currently, the development of prosocial behavior has become an important indicator for measuring the level of socialization among children and adolescents (Li et al., 2025). The junior high school stage serves as a critical period for socialization development. Cultivating prosocial behavior during this phase helps advance the process of adolescents' socialization development (Gao et al., 2021).

The concept of prosocial behavior was first proposed by Weisperg in 1972. It primarily refers to the collective term for actions individuals take that benefit others, groups, or society as a whole. This term contrasts with antisocial behaviors such as violence and aggression, as prosocial actions are undertaken to benefit others and society (Wispe, 1972). In the development of this research field, there remains no consensus on the definition of its concepts, with the primary disagreement centering on the motives of those implementing it. Myerswrited posits that prosocial behavior constitutes a process through which individuals achieve self-satisfaction, incorporating elements of guilt avoidance and negative mood regulation (personal distress, negative emotions), potentially representing self-oriented altruism (Myers, 1990). Weerdmeester defines prosocial behavior as actions motivated by altruism, characterized by full consideration for others without seeking reward, and potentially involving personal sacrifice in the process (Weerdmeester and Lange, 2019). Regardless of the motivation behind it, prosocial behavior consistently yields outcomes that align with societal expectations and benefit others and society at large (García-García et al., 2020). Furthermore, developmental psychologist Nancy Eisenberg, through her systematic research, has particularly emphasized that adolescence is a critical stage for the development of prosocial behavior. Her proposed prosocial moral reasoning model indicates that individuals‘ prosocial behaviors are closely linked to their level of moral reasoning. As cognitive abilities develop, adolescents' prosocial motivations gradually shift from self-centered orientations (such as seeking rewards or avoiding punishment) toward more advanced other-oriented and principle-based orientations. (Eisenberg, 1986; Eisenberg et al., 2006) This theoretical framework provides an important theoretical foundation for understanding how exercise interventions promote prosocial behavior among junior high school students by influencing their cognitive and emotional processes.

Prosocial behavior undergoes significant development and change during adolescence, influenced by the combined effects of individual traits, subjective experiences, and collective environments. (Seligman et al., 2006). Physical exercise can positively impact an individual's physical and mental health by fostering positive personality traits, enhancing subjective wellbeing, and creating a supportive group atmosphere. Research indicates that physical exercise is positively correlated with an individual's prosocial behavior (Jiang and Bian, 2025). Individuals who regularly engage in physical exercise exhibit more helping, sharing, and cooperative behaviors. Therefore, physical exercise may provide motivation across these three levels, thereby influencing prosocial behavior. Furthermore, interaction ritual theory posits that a successful interaction ritual requires four key elements: group gathering, exclusion of outsiders, a shared focal point, and a shared emotional state. When these elements are effectively combined and mutually reinforced, they give rise to four core outcomes: group solidarity, individual emotional energy, social relational symbols, and shared moral standards (Jiang and He, 2016). Physical exercise, as a quintessential interactive ritual (Zhang et al., 2021), fosters emotional connection and identity recognition among participants through ritualized physical activities, thereby exerting positive effects on individuals‘ psychological states and social behaviors. However, physical exercise is like a double-edged sword: while offering numerous benefits, it also carries risks that cannot be ignored. Particularly in competitive sports, without proper guidance in values, it may instead foster antisocial tendencies such as aggressive behavior, cheating, and verbal abuse. Existing research indicates that such behavior is closely associated with individuals' inadequate emotional regulation skills, an overly self-centered view of winning and losing, and a weak sense of sportsmanship (Kavussanu and Boardley, 2009; Hodge and Gucciardi, 2015). Therefore, how to design scientific interventions to mitigate potential risks in sports while maximizing their positive benefits for social development has become a critical issue. This study argues that not all physical education practices automatically promote positive development. Only when physical education interventions are consciously structured within a value framework emphasizing cooperation, respect, empathy, and fair play can they effectively curb antisocial tendencies and robustly cultivate prosocial behaviors (García-González et al., 2023).

There may be additional mediating variables involved in the process by which physical exercise exerts a positive influence on prosocial behavior. In psychological research, empathy is a complex, multifaceted psychological process—the ability of an individual to understand another person's emotions and feelings and respond appropriately (Baron-Cohen and Wheelwright, 2003). Research indicates that empathy can promote prosocial behavior in individuals (Javed et al., 2025). According to Batson's empathy-altruism hypothesis model, prosocial behavior is primarily triggered by empathy. When others face distress, it elicits an empathetic response in individuals, generating altruistic motivation to alleviate their suffering, thereby prompting prosocial actions (Batson, 1987). Therefore, empathy, as a key motivational source for promoting prosocial behavior (Luberto et al., 2018), plays a significant role in the emergence of an individual's prosocial actions.

Empathy is the core ability for individuals to understand and share others‘ feelings (Sodoma and Sharp, 2025), with its neural basis primarily relying on the coordinated activity of specific brain regions, including the anterior insula, sensorimotor cortex, and supplementary motor area (Levy et al., 2019). Research indicates that physical exercise is significantly positively correlated with empathy (Man et al., 2022). The physiological mechanism may be that physical exercise effectively activates the neural systems associated with empathy (Xu et al., 2020), enhancing functional connectivity and responsiveness in the aforementioned brain regions, thereby promoting the development of empathy. Furthermore, according to ritual interaction theory, maintaining a high degree of mutual attention during ritual interactions—accompanied by close physical interaction and synchronized bodily rhythms—can mutually evoke participants' emotions and feelings. This fosters a strong emotional bond, generating greater emotional energy for participants and enhancing their passion and willingness to engage in behavior aligned with ethical standards (Jiang and He, 2016). Therefore, empathy may mediate the relationship between physical exercise and prosocial behavior.

On the other hand, interpersonal relationships refer to the psychological distance and connection formed between individuals during mutual interactions, reflecting the psychological state of individuals or groups seeking to satisfy their needs (Moldes, 2025). Research indicates that prosocial behavior occurs within interpersonal interactions, and positive relationships can foster the emergence of such behavior (Fitzsimons and Bargh, 2003). Individuals with strong interpersonal skills are more likely to initiate and maintain communication with others. During interactions, they demonstrate greater friendliness, offer more support, and are able to resolve conflicts calmly (Rismayadi, 2024). The mechanism may be that when an interpersonal relationship object is activated, the goal of strengthening and maintaining that relationship becomes the individual's behavioral orientation, subconsciously influencing their social behavior (Fitzsimons and Bargh, 2003).

Interpersonal relationships are established and developed through mutual interaction between individuals. Physical exercise provides a platform for individuals to engage with one another, enhancing interpersonal communication and creating more opportunities for social interaction, thereby effectively improving one's interpersonal relationships (Martín-Rodríguez et al., 2024). Research indicates that adolescents who actively engage in physical exercise are better at communicating emotions and sharing enjoyment with others (Yan et al., 2019), effectively resolving interpersonal conflicts, and developing stronger relationships (Zhang, 2016). Furthermore, according to ritual interaction theory, maintaining a high degree of mutual attention during a ritual interaction—accompanied by close physical interaction and synchronized bodily rhythms—can evoke participants' emotions and feelings, forming a strong emotional bond. This creates a sense of membership tied to cognitive symbols, thereby increasing their passion and desire to engage in behavior aligned with moral standards (Jiang and He, 2016). Therefore, interpersonal relationships may mediate the relationship between physical exercise and prosocial behavior.

Symbolic interaction theory posits that to successfully integrate into their social environment, individuals internalize typical interaction symbols widely recognized in interpersonal relationships (Zhu F. et al., 2022). They interpret their relationships with others and their respective roles based on these symbols, thereby engaging in interactions that align with societal expectations (Baldwin, 1992). Within this theoretical framework, empathy can be viewed as a crucial symbolic interaction process: the empathizer conveys relational signals to the other by understanding their emotions and responding appropriately in line with role expectations; the recipient of empathy, in turn, clarifies their own role within the relationship and its social implications by receiving and interpreting these symbols, subsequently responding with behavior consistent with the expectations of that role. In this ongoing cycle of symbolic exchange, both parties continually adjust their behavioral expectations and interaction patterns, thereby gradually deepening and solidifying interpersonal relationships. Therefore, empathy and interpersonal relationships may serve as chain mediators between physical exercise and prosocial behavior.

The Special Action Plan for Comprehensively Strengthening and Improving Student Mental Health Work in the New Era (2023–2025) states that physical education should be leveraged to regulate emotions and alleviate stress. Efforts should focus on ensuring students engage in one hour of physical activity both on and off campus daily, master one to two sports skills proficiently, and derive enjoyment from exercise while enhancing physical fitness, developing sound character, and strengthening willpower. (Fan et al., 2023) Compared to individual sports, team sports are more conducive to cultivating students' behavioral qualities such as cooperation, sharing, and mutual assistance. Basketball, as a quintessential team sport, has been incorporated into the “Compulsory Education Physical Education and Health Curriculum Standards (2022 Edition).” It requires junior high school students to master basic skills. Given its widespread availability of playing facilities and strong foundation of student participation, the sport enjoys broad applicability within school settings. Numerous studies have confirmed that basketball-based physical interventions positively impact the mental health of junior high school students. Such programs promote physical and mental wellbeing, enhance interpersonal communication and teamwork skills (Xiong, 2023), and help cultivate strong willpower and a spirit of collectivism (Zhang et al., 2010). Therefore, selecting basketball as the subject of the sports intervention experiment demonstrates strong universality and practical feasibility.

In summary, the following hypotheses are proposed:

H1: Exercise intervention can enhance empathy, interpersonal relationships, and prosocial behavior among junior high school students.

H2: Exercise intervention can indirectly influence prosocial behavior through empathy and interpersonal relationships, respectively.

H3: Empathy and interpersonal relationships play a chain-mediated role in the exercise intervention's enhancement of prosocial behavior.

## 2 Methods

### 2.1 Procedure and participants

This experimental study was conducted at a secondary school in Zhenjiang City, Jiangsu Province, China, and was approved by the Ethics Committee of Yangzhou University Medical College (YXYLL-2025–124). Data collection before and after the experiment was conducted through offline questionnaires with the assistance of school administrators. Participants completed the questionnaire on-site under standardized guidance and returned it immediately. All students signed informed consent forms prior to participation. Inclusion criteria are: (1) no recent severe physical illness; (2) frequent daily physical activity; (3) presence of mental health. After screening the collected questionnaires, samples exhibiting highly consistent response patterns or incomplete answers were excluded.

Using G-Power 3.1 software to estimate the sample size, it was determined that a total sample size of at least 86 participants is required to ensure 80% statistical power (1-β power) and an effect size of 0.25. Therefore, two classes of first-year junior high school students totaling 98 individuals were selected from a certain secondary school as the initial experimental subjects using a cluster sampling method. Based on the Physical Activity Level Scale and the Chinese Adolescent Mental Health Scale, six students were excluded due to frequent daily physical activity or existing mental health issues. Additionally, two students withdrew from the experiment midway due to physical discomfort. A total of 90 first-year junior high students (42 boys and 48 girls) from two classes were ultimately selected as subjects for the experimental study. The class was randomly divided into an experimental group and a control group using a lottery method. The experimental group comprised 45 students (20 males and 25 females, with an average age of 13.33 ± 0.52 years), while the control group included 45 students (22 males and 23 females, with an average age of 13.16 ± 0.48 years). The athletic backgrounds of all participants align with the typical profile for adolescents in this age group, meaning they primarily engage in physical exercise through school physical education classes and have not undergone specific selection processes for basketball programs.

### 2.2 Experimental design

To clarify the scope of this study and avoid conceptual confusion, the core concepts are defined according to the standards of the American College of Sports Medicine: physical activity: any bodily movement produced by skeletal muscles that results in energy expenditure; Exercise: planned, organized, and repeated physical activity performed with the goal of maintaining or improving physical fitness; Sports: refers to competitive physical activities conducted under a set of established rules, typically for recreational or professional purposes. In this study, the control group's conventional physical education intervention included exercises aimed at skill acquisition and health promotion, as well as a variety of physical activities. The basketball intervention in the experimental group specifically refers to a team sport that not only provides systematic physical exercise but also possesses distinct social structural characteristics such as clear rules, competitiveness, teamwork, and strategic collaboration.

A 2 (group: 1 experimental group, 1 control group) × 2 (Time: Pre-test, Post-test) mixed-design experimental study. The independent variables were basketball-based intervention (experimental group) and conventional physical education class intervention (control group). The dependent variable was prosocial behavior among junior high school students, with empathy and interpersonal relationships serving as mediating variables. This natural experiment examined both between-group and within-group pre- and post-test comparisons between the experimental and control groups.

The experimental group underwent a 12-week basketball intervention program consisting of three sessions per week, each lasting 30 min at moderate-to-vigorous intensity (excluding warm-up and cool-down activities). The essence of basketball intervention lies in “values-guided physical education,” whose core principle is to organically integrate value formation into the learning of athletic skills. The entire intervention process is guided by the following four key operational points and their corresponding principles: (1). Structured Collaborative Tasks: by establishing specific rules, these tasks reinforce cooperation and team spirit, guiding students to experience firsthand how individual contributions advance shared team objectives. (2). Emotional Regulation Guidance: at critical moments during competitions, teachers intervene promptly to guide students in identifying emotions such as frustration. They demonstrate strategies like positive self-talk, emphasizing the cultivation of emotional recognition and regulation skills. (3). Reinforcing Sportsmanship: clearly stipulate and immediately commend actions such as helping opponents up and respecting referees. Through prompt discussions, internalize the value of these behaviors, thereby embedding them into sportsmanship education. (4). Role-Playing and Empathy Discussion: utilize post-game role-swapping and sharing sessions to create empathetic scenarios, encouraging students to adopt different perspectives and deepen their understanding of teammates and opponents. The selection of exercise intensity is based on moderate to vigorous intensity as proposed by the Chinese Health Physical Education Curriculum Model (Ji, 2015). Prior to each basketball intervention session, 30 randomly selected participants wore Polar heart rate monitors to track exercise intensity, with moderate-to-vigorous intensity maintained at 140–160 beats per min.

The control group underwent a 12-week conventional physical education intervention guided by the experimental school's curriculum plan, covering activities such as gymnastics, martial arts, table tennis, and physical fitness training. The frequency, duration, and intensity of classes were identical to those of the experimental group, but no systematic emphasis or guidance was provided regarding the aforementioned values and ethical principles.

Both groups of students received instruction from the same teacher at fixed times and locations. Teaching methods and organizational formats were diversified according to the characteristics of the sports activities, creating complex movement scenarios through group cooperation, competitive drills, and matches.

### 2.3 Research tools

#### 2.3.1 Physical activity level scale

The Physical Activity Level Scale developed by Hashimoto and revised by Liang was used for assessment (Liang, 1994). This scale calculates activity levels by integrating exercise intensity, duration, and frequency using the formula “Intensity × (Duration–1) × Frequency.” The total score ranges from 0 to 100 points, defining three activity levels: low (≤ 19 points), moderate (20–42 points), and high (≥43 points). In this study, the Cronbach's α coefficient for this scale was 0.760.

#### 2.3.2 Chinese adolescent mental health scale

The Chinese Adolescent Mental Health Scale developed by Wang Jisheng et al. was employed (Wang et al., 1997). This scale comprises 60 items scored on a 5-point scale. The total mean score across all items was calculated to assess overall mental health status. A total mean score ≥2 was considered abnormal, with severity categorized as follows: mild (2–2.99), moderate (3–3.99), relatively severe (4–4.99), and severe (5). In this study, the Cronbach's α coefficient for this scale was 0.979.

#### 2.3.3 Basic empathy scale

The Basic Empathy Scale translated by Li Chenfeng et al. was adopted (Li et al., 2011). This scale comprises 20 items across two dimensions, scored on a 5-point scale, with higher scores indicating stronger empathy abilities among participants. In this study, the scale demonstrated good reliability and validity, with a Cronbach's α coefficient of 0.776, X^2^/df = 1.224, RMSEA = 0.035, CFI = 0.952, TLI = 0.937, and SRMR = 0.042.

#### 2.3.4 Interpersonal relationship diagnostic scale

The Interpersonal Relationship Diagnostic Scale developed by Zheng et al. was employed (Chen and Zhang, 2021), comprising 28 items across 4 dimensions. The total score on this scale serves as a key indicator for assessing the quality of an individual's interpersonal relationships. A lower score indicates higher relationship quality and fewer interpersonal difficulties. Specifically, a total score between 0 and 8 indicates harmonious interpersonal relationships with no interpersonal distress; a score between 9 and 14 indicates mild interpersonal distress, suggesting room for improvement in relationship quality; and a score between 15 and 28 indicates more severe interpersonal distress, reflecting poor relationship quality. In this study, the scale demonstrated good reliability and validity, with a Cronbach's α coefficient of 0.860, X^2^/df = 1.135, RMSEA = 0.027, CFI = 0.929, TLI = 0.910, and SRMR = 0.072.

#### 2.3.5 Prosocial behavior scale

The Prosocial Behavior Tendency Scale developed by Carroll and revised by Kou Yu et al. was employed (Kou et al., 2007). This 26-item scale uses a 5-point scoring system, with higher total scores indicating greater prosocial behavior tendencies among participants. In this study, the Cronbach's α coefficient for this scale was 0.910, with X^2^/df = 1.468, RMSEA = 0.051, CFI = 0.927, TLI = 0.910, and SRMR = 0.067. These results indicate that the scale possesses good reliability and validity.

### 2.4 Data processing

Data processing was conducted using SPSS 26.0 and AMOS 24.0, specifically including: SPSS was used for reliability analysis, Harman's single-factor test (common method bias), normality tests and corresponding homogeneity tests (*t*-tests or Mann-Whitney U tests), repeated measures and one-way ANOVA (within- and between-group differences), bivariate correlation analysis, multicollinearity tests, and finally the Process macro for mediating effect testing (Hayes, 2013).

## 3 Results

### 3.1 Common method bias test

This study employed self-report scales to measure empathy, interpersonal relationships, and prosocial behavior among participants, which may introduce common method bias. A common method bias test using Harman's single-factor method was conducted. The results revealed 24 common factors with eigenvalues greater than 1, with the first common factor explaining 16.56% of the variance (below the critical threshold of 40%). Therefore, this study does not exhibit significant common method bias.

### 3.2 Descriptive statistics of demographic variables for students in different groups

A normality test was conducted on the pre-test data, revealing that the empathy, interpersonal relationships, and prosocial behaviors of junior high school students all followed a normal distribution. Therefore, an independent samples *t*-test was employed to analyze the homogeneity of the variables. Prior to the commencement of the exercise intervention, an independent samples *t*-test was conducted on the two groups of students regarding demographic variables (gender, age, height, weight) and physical activity levels. The results indicated that there were no significant differences between the experimental group and the control group across all indicators, demonstrating homogeneity.

### 3.3 Pre-intervention homogeneity and post-intervention between-group difference test

To examine the homogeneity between the experimental and control groups prior to intervention and to assess the immediate effects following intervention, this study conducted independent samples *t*-tests on both pretest and posttest data. The results indicate (see Table 1) that prior to the experiment, there were no statistically significant differences between the two groups of junior high school students in terms of the levels of each variable (p > 0.05). The results indicate that prior to the experiment, the two groups of junior high school students exhibited comparable levels of empathy, interpersonal relationships, and prosocial behavior, making them suitable for subsequent comparative analysis. After 12 weeks of intervention, significant changes occurred in the between-group comparison results. The main effect of group reached statistical significance across all variables.

**Table 1:** Pre- and post-test scores of the experimental and control groups and between-group difference tests.

**Variable**	**Time**	**Experimental group**	**Control group**	**Intergroup differences**		
		**M** ±**SD**	**M** ±**SD**	***t***	***p***	***Cohen's d***
Empathy	Pre-test	68.27 ± 7.18	69.64 ± 10.77	0.71	0.47	−0.15
	Post-test	79.56 ± 8.05	73.44 ± 11.14	−2.98	<0.05	0.63
Interpersonal relationships	Pre-test	11.76 ± 2.84	12.78 ± 2.76	−1.73	0.08	−0.36
	Post-test	6.98 ± 2.14	9.27 ± 3.19	4.00	<0.001	−0.84
Prosocial behavior	Pre-test	90.24 ± 15.36	87.00 ± 13.39	−1.07	0.29	0.22
	Post-test	108.11 ± 7.94	95.53 ± 13.61	−5.35	<0.001	1.13

M denotes the mean, SD denotes the standard deviation. Lower interpersonal relationship scores indicate better relationship quality. A positive Cohen's d value indicates that the experimental group scored higher than the control group, while a negative value indicates that the experimental group scored lower than the control group.

### 3.4 Effects of exercise intervention on middle school students' levels of empathy, interpersonal relationships, and prosocial behavior

This study employed a 2 (group: experimental/control) × 2 (time: pretest/posttest) repeated measures analysis of variance to examine the effects of group and time on middle school students' empathy, interpersonal relationships, and prosocial behavior. The results (see Table 2) indicate that the main effect of time was significant: empathy, interpersonal relationships, and prosocial behavior all exhibited significant changes over time. Regarding the main effects of group, neither empathy nor interpersonal relationships showed significant effects. Only prosocial behavior showed significant between-group differences. The time × group interaction was significant: empathy, interpersonal relationships, and prosocial behavior all indicated significant differences in the temporal trends between the two groups.

**Table 2:** Analysis of variance for the effects of exercise intervention on various variable levels among junior high school students.

**Variable**	**Source of mutation**	**Class III sum of squares**	**Degree of freedom**	**Mean square**	***F***	***p***	***η^2^p***
Empathy	Time	2561.339	1	2561.339	319.16	<0.01	0.784
	Error	706.222	88	8.025			
	Group	252.050	1	252.050	1.48	0.227	0.017
	Error	14985.111	88	164.73			
	Time × group	630.939	1	2347.18	78.62	<0.01	0.472
Interpersonal relationships	Time	772.939	1	772.939	265.47	<0.01	0.751
	Error	256.222	88	2.912			
	Group	18.050	1	18.050	1.47	0.229	0.016
	error	1081.644	88	12.291			
	Time × group	123.339	1	123.339	42.36	<0.01	0.325
Prosocial behavior	Time	7840.8	1	7840.8	95.06	<0.01	0.524
	error	7109.2	88	80.786			
	Group	2816.356	1	2816.356	11.21	<0.01	0.113
	error	22094.756	88	251.077			
	Time × group	980.000	1	980.000	12.13	<0.01	0.121

After conducting a simple effect analysis (Table 3), it was found that prior to the experiment, there were no statistically significant differences among the different groups of junior high school students in terms of empathy, interpersonal relationships, or prosocial behavior. At post-experiment assessment, statistically significant differences were observed across different groups of junior high school students in empathy, interpersonal relationships, and prosocial behavior. Under the experimental group conditions, significant statistical differences were observed in junior high students‘ empathy, interpersonal relationships, and prosocial behavior across different time factors. Under control group conditions, significant statistical differences were observed in junior high students' interpersonal relationships and prosocial behaviors across different time factors, while no significant statistical differences were found in empathy.

**Table 3:** Simple effects analysis of the interaction between time and group on variable levels among junior high school students.

**Variable**	**Simple main effect**	**Sum of squares**	**Degree of freedom**	**Mean square**	***F***	***p***	***η^2^p***
Empathy	Pre-test	42.711	1	42.711	4.79	0.49	0.003
	Error	15691.333	176	89.155			
	Post-test	840.278	1	840.278	9.43	<0.01	0.051
	error	15691.333	176	89.155			
	Experimental group	2867.378	1	2867.378	32.16	<0.01	0.020
	Error	15691.333	176	89.155			
	Control group	324.900	1	324.900	3.64	0.058	0.155
	Error	15691.333	176	89.155			
Interpersonal Relationships	Pre-test	23.511	1	23.511	3.09	0.08	0.017
	Error	1337.867	176	7.602			
	Post-test	117.878	1	117.878	15.51	<0.01	0.081
	Error	1337.867	176	7.602			
	Experimental group	756.900	1	756.900	99.57	<0.01	0.361
	Error	1337.867	176	7.602			
	Control group	139.378	1	139.378	18.34	0.01	0.094
	Error	15691.333	176	89.155			
Prosocial behavior	Pre-test	236.844	1	236.844	1.43	0.23	0.008
	error	29203.956	176	165.932			
	Post-test	3559.511	1	3559.511	21.45	<0.01	0.109
	Error	2903.956	176	165.932			
	Experimental group	7182.400	1	7182.400	43.29	<0.01	0.197
	error	29203.956	176	165.932			
	Control group	1638.400	1	1638.400	9.87	0.02	0.053
	Error	29870.19	176	164.12			

**Figure 1:**
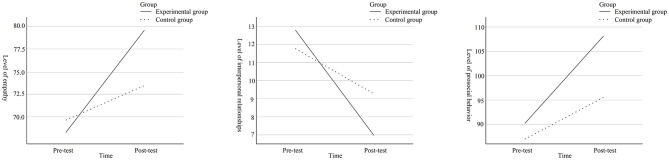
Changes in variable levels across groups before and after the experiment.

Multiple comparison results and the simple slope diagram (see Figure 1) indicate that after 12 weeks of exercise intervention, post-test levels of all variables were higher in the experimental group than in the control group among junior high school students. However, the improvement in empathy levels among control group students was not statistically significant. The experimental group of junior high school students demonstrated significantly higher levels across all variables compared to the control group, and the intervention effect was also superior to that of the control group. This result supports Hypothesis H1.

### 3.5 Testing intervention effects based on multivariate covariance analysis

To more accurately assess the net effect of the basketball intervention, this study conducted a multivariate analysis of covariance after controlling for baseline levels. Post-test empathy, post-test interpersonal relationships, and post-test prosocial behavior were each used as dependent variables, with group assignment serving as the independent variable. Simultaneously, the scores from all three pre-tests were incorporated into the model as covariates. The results indicate (see Table 4) that the main effect of group was significant for all posttest dependent variables (*p* < 0.001), with effect sizes (η^2^*p*) ranging from 0.327 to 0.499, falling within the large effect range.

**Table 4:** Multivariate analysis of covariance with each post-test variable as the dependent variable.

**Variable**	**Source of mutation**	**Class III sum of squares**	**Degree of freedom**	**Mean square**	***F***	***p***	***η^2^p***
Empathy	Pre-test	4406.460	1	4406.460	286.898	<0.001	0.771
	Group	1300.967	1	1300.967	84.704	<0.001	0.499
	Error	1305.515	85	15.359			
Interpersonal relationships	Pre-test	64.999	1	64.999	17.850	<0.001	0.174
	Group	150.660	1	150.660	41.375	<0.001	0.327
	Error	309.515	85	3.641			
Prosocial behavior	Pre-test	1525.982	1	1525.982	30.384	<0.001	0.263
	Group	4182.836	1	4182.836	83.284	<0.001	0.495
	error	4269.024	85	50.224			

### 3.6 Correlation analysis of exercise intervention, empathy, interpersonal relationships, and prosocial behavior levels

Using correlation analysis to examine the relationships among basketball intervention, empathy, interpersonal relationships, and prosocial behavior among junior high school students, the results indicate (Table 5) that significant correlations exist between basketball intervention, empathy, interpersonal relationships, and prosocial behavior (*p* < 0.01). Therefore, further analysis can be conducted.

**Table 5:** Correlation analysis among variables (post-test).

**Variable**	**Exercise intervention**	**Empathy**	**Interpersonal relationships**	**Prosocial behavior**
**Exercise intervention**				
Empathy	0.303^**^			
Interpersonal relationships	−0.392^**^	−0.623^**^		
Prosocial behavior	0.496^**^	0.741^**^	−0.830^**^	

^*^means that *p*-value is <0.05; ^**^means that *p*-value is <0.01; ^***^means that *p*-value is <0.001.

### 3.7 Test for multicollinearity

Correlation analysis indicated significant associations among variables, prompting a multicollinearity test. Results showed tolerance values for all predictor variables ranging from 0.68 to 0.75 (greater than 0.1), with variance inflation factors (VIF) between 1.33 and 1.48 (less than 5). Therefore, the data in this study do not exhibit multicollinearity issues, allowing for the subsequent examination of mediating effects and chained mediating effects.

### 3.8 Model testing of the effects of exercise interventions on prosocial behavior in junior high school students

This study employed the SPSS macro program Process developed by Hayes to construct and test a bootstrap-based chained mediation model (Hayes, 2013). Model 6 was selected for chained mediation testing, with group membership as the independent variable, empathy and interpersonal relationships as mediating variables, and prosocial behavior as the dependent variable. After standardizing all variables, the analysis was conducted. The results showed (Table 6) that the entire regression equation was significant (*R*^2^ = 0.25, *F* = 28.66, *p* < 0.01), with the effect size of the basketball intervention on prosocial behavior being 0.49.

**Table 6:** Regression analysis of the chain mediation model for empathy and interpersonal relationships (post-test/standardized).

**Variable**	**Empathy**		**Interpersonal relationships**		**Prosocial behavior**		**Overall effect**	
	β	***t***	β	***t***	β	***t***	β	***t***
Exercise intervention	0.30	2.98^**^	−0.22	−2.64^**^	0.18	3.34^**^	0.49	5.35^***^
Empathy			−0.56	−6.56^***^	0.35	5.59^***^		
Interpersonal relationships					−0.54	−8.39^***^		
Prosocial behavior								
Control variables								
Age	−0.61	−2.72^**^	0.06	0.42	−0.04	−0.34		
Gender	−0.22	−0.97	−0.02	−0.14	0.01	0.10		
*R^2^*	0.09		0.43		0.80		0.25	
*F*	8.89^**^		33.38^**^		112.11^**^		28.66^**^	

^*^means that *p*-value is <0.05; ^**^means that *p*-value is <0.01; ^***^means that *p*-value is <0.001.

**Figure 2:**
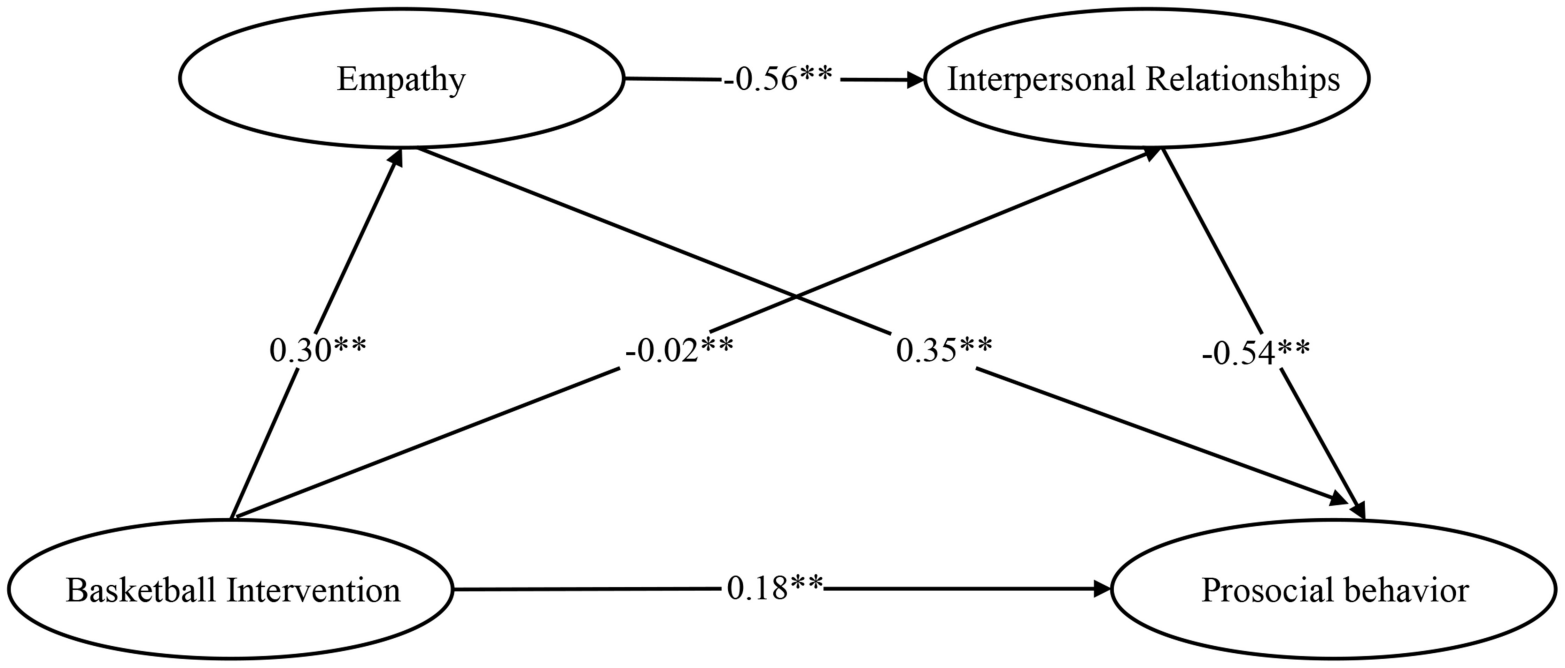
Chain mediation model of empathy and interpersonal relationships between exercise intervention and prosocial behavior.

The results of the regression analysis (Table 6) indicate that the basketball intervention positively influenced empathy, interpersonal relationships, and prosocial behavior (β = 0.30, *t* = 2.98, *p* < 0.01; β = −0.22, *t* = −2.64, *p* < 0.01; β = 0.18, *t* = 3.34, *p* < 0.01); Empathy significantly enhances interpersonal relationships and prosocial behavior (β = −0.56, *t* = −6.56, *p* < 0.01; β = 0.35, *t* = 5.59, *p* < 0.01); Interpersonal relationships significantly enhance prosocial behavior (β = −0.54, *t* = −8.39, *p* < 0.01). Based on the above findings, a chained mediation effect path testing model diagram concerning empathy and interpersonal relationships was established (Figure 2).

The results of the mediation model effect analysis (Figure 2 and Table 7) indicate that the direct effect of the exercise intervention on prosocial behavior is significant, with an effect size of 0.177 (Bootstrap CI [0.176–0.471]) and a relative effect proportion of 35.71%. The total indirect effect size across all pathways is 0.213 (Bootstrap CI [0.080–0.382]), accounting for 64.29% of the relative effect. Among these, indirect effect 1 (exercise intervention → empathy → prosocial behavior) was significant, with an effect size of 0.106 (Bootstrap CI [0.035–0.212]) and a relative effect proportion of 21.33%; Indirect effect 2 (exercise intervention → interpersonal relationships → prosocial behavior) was significant, with an effect size of 0.122 (Bootstrap CI [0.014–0.236]) and a relative effect proportion of 24.54%. Indirect effect 3 (exercise intervention → empathy → interpersonal relationships → prosocial behavior) was significant, with an effect size of 0.091 (Bootstrap CI [0.035–0.163]) and a relative effect contribution of 18.42%. Therefore, the chain mediation effect of empathy and interpersonal relationships in the positive effects of basketball-based sports interventions on prosocial behavior is established, validating Hypothesis 2 and Hypothesis 3.

**Table 7:** Testing the chain mediation model effects of exercise intervention on empathy, interpersonal relationships, and prosocial behavior.

**Effect type**	**Effect size**	***BootSE***	***Bootstrap 95%CI***		**Relative effect contribution**
			**Lower limit**	**Upper limit**	
Overall effect	0.496	0.093	0.312	0.679	100%
Direct effect	0.177	0.053	0.176	0.471	35.71%
Indirect effect 1	0.106	0.045	0.035	0.212	21.33%
Indirect effect 2	0.122	0.057	0.014	0.236	24.54%
Indirect effect 3	0.091	0.040	0.035	0.163	18.42%
Total indirect effect	0.213	0.077	0.080	0.382	64.29%

Indirect Effect 1, Basketball Program Intervention → Empathy → Prosocial Behavior; Indirect Effect 2, Basketball Program Intervention → Interpersonal Relationships → Prosocial Behavior; Indirect Effect 3, Basketball Program Intervention → Empathy → Interpersonal Relationships → Prosocial Behavior.

## 4 Discussion

The results of this study indicate that both the experimental and control groups of junior high school students demonstrated improvements across all variables. Specifically, the experimental group showed significant increases in empathy, interpersonal relationships, and prosocial behavior, while the control group exhibited significant improvements in interpersonal relationships and prosocial behavior. Although the control group's empathy levels increased to some extent, the improvement did not reach statistical significance. The aforementioned changes may be attributed to the scientifically designed exercise program developed by this research institute, which incorporates optimal configurations in terms of intensity, duration, frequency, and cycle. Existing research indicates that physical exercise has a positive impact on adolescents‘ mental health, specifically manifested in the following aspects: intensity: moderate-to-vigorous exercise maintaining an average heart rate of 140–160 beats per min yields superior effects in enhancing mental health, sports ethics, interpersonal relationships, and healthy behaviors (Zhang et al., 2022; Ji, 2022). Regarding duration: moderate-to-vigorous exercise lasting 31–59 min yields greater mental health benefits than shorter sessions (Zhang, 2016), but exceeding an individual's tolerance threshold may produce negative effects due to dose-response dynamics (Salmon, 2001). Regarding frequency: exercising 3–5 times per week for 30–60 min per session yields the most positive benefits for mental health. Excessive frequency or overly long single sessions may actually be detrimental to mental wellbeing (Chekroud et al., 2018). In terms of duration: the minimum effective period for physical exercise to produce psychological benefits is 10–12 weeks (Zeng et al., 2021). This study protocol was designed as a 12-week program involving moderate-to-vigorous intensity exercise three times per week for 30 min per session, aligning with the aforementioned characteristics of effective interventions. Consequently, it produced positive psychological benefits for both groups of students. The experimental group showed significantly greater improvements across all variables compared to the control group, which may be related to the nature of the exercise program. The control group involved exclusively individual sports, which emphasize self-referentiality and intrinsic motivation but lack deep-level interaction and collaboration. This approach is not conducive to fostering teamwork and collective consciousness. Basketball, as a team sport that combines competition and cooperation, effectively sparks students' interest and promotes positive transfer in their cognitive, emotional, relational, and behavioral domains, thereby delivering more comprehensive psychosocial benefits. Although the control group's regular physical education classes also included physical activities and cooperative segments, their effects on promoting empathy, interpersonal relationships, and prosocial behavior were significantly lower than those of the experimental group. This finding strongly suggests that the psychosocial benefits derived from physical activity depend largely on the “process” and “atmosphere” of program implementation—namely, whether they are grounded in a clear ethical framework and value-based guidance. The basketball intervention program implemented in the experimental group of this study successfully transformed competitive physical activity into a social learning platform by systematically integrating teamwork, emotional regulation, and sports ethics education. In such an environment characterized by value-driven guidance, students not only develop physical fitness but also learn how to cope with setbacks in competitive situations (thereby preventing antisocial behavior) and how to understand others and collaborate effectively (thereby promoting prosocial behavior).

### 4.1 Effects of exercise intervention on empathy levels among junior high school students.

Empathy, as the foundation of interpersonal interaction and social adaptation, manifests not only as the understanding and sharing of others' feelings (Sodoma and Sharp, 2025), but also possesses a distinct neurophysiological basis supported by neural networks comprising brain regions such as the anterior insula and sensorimotor cortex (Levy et al., 2019). Research indicates that physical exercise activates this network, enhances functional connectivity within brain regions, and thereby improves empathy capacity (Xu et al., 2020). From the perspective of social interaction, interactional ritual theory posits that individuals synchronizing body language and rhythms within shared contexts can evoke emotional responses and foster emotional bonds (Jiang and He, 2016). Physical exercise, as a quintessential interactive ritual, provides participants with an embodied platform for interaction. During this process, individuals enhance emotional resonance and induce empathy through bodily-cognitive integration experiences such as movement coordination, intention prediction, and rhythmic adaptation (Song and Dong, 2023).

After 12 weeks of experimentation, the empathy levels of junior high students in the control group showed no significant improvement. This may be attributed to the conventional physical education curriculum, which involves a wide range of sports activities and primarily focuses on individual sports. For middle school students, the content appears monotonous and tedious, making it difficult to sustain their enthusiasm for participation. During this process, opportunities for communication and collaborative interaction among students have correspondingly decreased, which has to some extent diminished their interest in physical activity. Positive interest in physical activity promotes the development of an individual's empathy skills (Kalliopuska, 1987). The experimental group of junior high students demonstrated a significant improvement in empathy levels. First, basketball is a highly competitive and comprehensive sport. By incorporating combination techniques into instruction, students must coordinate movements, anticipate game situations, and continuously interact with teammates during competitive drills and simulated matches. This cognitive-behavioral integration within complex scenarios promotes the development of empathy skills. Second, as a team sport, basketball relies on communication, trust, and collaboration among teammates. By creating diverse cooperative scenarios, the experiment enhanced understanding and coordination among students, providing a social foundation for the development of empathy. Third, tactical instruction is integrated throughout the experiment to cultivate students' ability to observe and anticipate the intentions of teammates and opponents. This enhances their cognitive understanding and role immersion in dynamic scenarios, thereby supporting the development of empathy.

### 4.2 Effects of exercise intervention on interpersonal relationship levels among junior high school students.

The theoretical model of psychological benefits from youth sports activities posits that physical exercise can directly influence adolescents' behavioral outcomes such as interpersonal interactions (Tomporowski et al., 2011). Specifically, sports activities provide individuals with platforms for mutual exchange, thereby enhancing interpersonal interactions and improving the quality of relationships (Lee et al., 2021). From the perspective of interactive ritual theory, participants develop a high degree of mutual attention and emotional resonance through frequent interactions, thereby establishing strong emotional bonds and forming a sense of membership linked to cognitive symbols (Jiang and He, 2016). Through participation in physical exercise, adolescents gain frequent opportunities for social interaction with others (Zhang, 2016). Within this theoretical framework, physical exercise serves as a positive social interaction ritual, creating a platform for middle school students to engage in social interactions with peers, teachers, and others. During exercise, students not only share the joy of physical activity but also form close emotional bonds through emotional exchange and shared feelings, thereby laying the foundation for building positive interpersonal relationships.

Carmichael's research found that compared to individual sports, team sports are more effective in enhancing adolescents' problem-solving abilities, promoting communication and cooperation skills, and positively guiding the development of their interpersonal relationships (Kelly, 2013). In this study, the basketball program adopted by the experimental group represents a quintessential team sport. Through daily instruction, practice sessions, and competitive matches, it provided junior high students with frequent opportunities for communication, interaction, collaboration, and competitive engagement. This enhanced their willingness to share and interact, strengthened their interpersonal skills, and laid the groundwork for building positive relationships. Furthermore, as a complex motor skill sport, basketball features diverse and dynamically evolving combinations of techniques and tactics. Particularly in game situations, participants must flexibly employ multiple strategies—such as communication and anticipation—to respond to the ever-changing demands of the court. At this point, their higher cognitive functions (cognitive control systems) become engaged, enhancing their capacity for empathy. This fosters mutual recognition and understanding, thereby developing interpersonal relationships between them (Zhu N. et al., 2022).

### 4.3 Effects of exercise intervention on prosocial behavior levels among junior high school students.

Prosocial behavior is influenced by an individual's positive traits, subjective positive experiences, and a collective positive environment. Physical exercise has the potential to provide positive support across these three dimensions, thereby promoting the formation and development of prosocial behavior (Seligman et al., 2006). According to symbolic interactionism, individuals engage in communication and reflection through symbols during social activities, thereby enhancing social cognition. People not only use symbols to understand the meaning of others' behaviors but also adjust their self-perception accordingly, evaluating how their own actions affect others. In this process, individuals continually refine their self-concept through feedback from others or the group, thereby guiding the implementation of subsequent behaviors (Liu, 2011). At the individual level, physical exercise provides participants with an interactive platform, increasing the frequency and depth of interpersonal interactions. Through sustained communication, individuals acquire self-image, behavioral norms, and social concepts, subsequently adjusting their own behavior based on perceived social expectations to develop prosocial tendencies aligned with societal norms (Lu, 2021). At the collective level, specific sports contexts can cultivate students' cooperative spirit, sense of dedication, and other positive qualities, which can then be transferred to their daily social lives, thereby promoting their social development (Li and Mao, 2015). From the perspective of subjective experience, sports competitions not only facilitate individuals' self-awareness and self-transcendence but also enhance their psychological resilience in coping with challenges and setbacks, thereby increasing their willingness and capacity for prosocial behavior (Lu, 2021). Additionally, the rules governing the competition process and the feedback on outcomes help students understand the consequences of their actions, develop an awareness of rules, and embrace mainstream values. This, in turn, enables them to regulate their own behavior and cultivate a stable prosocial orientation.

Team sports demonstrate a more pronounced effect than individual sports in enhancing students' prosocial behavior levels. As a quintessential team sport, basketball creates frequent opportunities for communication and interaction during practice and games, fostering greater social engagement among students. This interaction enables students to receive timely and clear feedback and evaluations of their own behavior from others, thereby facilitating reflection and self-adjustment. At the same time, basketball emphasizes teamwork, requiring students to collaborate toward shared goals during training and games, unconditionally fulfill collective tasks, curb self-centered tendencies, and learn to closely coordinate with teammates for the benefit of the group. This effectively cultivates middle school students' sense of collective responsibility and collaborative skills. Secondly, basketball is a highly competitive sport, particularly evident in game situations. By organizing systematic competitive drills and actual matches, the experimental group not only enhanced students' ability to comprehensively apply knowledge and skills to analyze and solve problems but also helped cultivate their sportsmanship, athletic character, and ethical conduct in sports (Ji, 2015). These qualities then transferred to social life, translating into more prosocial behaviors. Finally, throughout the teaching, practice, and competition phases of the experimental group, students were required to strictly adhere to the rules. This helped strengthen their awareness of rules, enabling them to learn to conform to mainstream value judgments and regulate their own behavior. Consequently, this sense of regulation extended to everyday situations, promoting the formation and development of prosocial behavior.

### 4.4 Chain mediation of empathy and interpersonal relationships.

Empathy is fundamentally a concept of “interpersonal relationships and connections” (Main et al., 2017). Simultaneously, as the foundation of successful interpersonal relationships, empathy plays a crucial role in facilitating individuals' ability to recognize others' emotions and establish and maintain relationships (Thompson et al., 2022). Research indicates that increased levels of empathy in individuals promote improvements in their interpersonal relationships (Zhang et al., 2023). This finding is consistent with the results of the present study. According to symbolic interaction theory, during social communication and interaction, the empathizer comprehends another person's emotions and responds appropriately, generating specific empathic symbols (such as facial expressions, language, and behaviors exhibited by the empathizer). The recipient of empathy receives and interprets these symbols, thereby clarifying their role implications within the relationship and the manner of connection with the other party. Subsequently, they respond with behaviors consistent with role expectations (Baldwin, 1992). In this ongoing process of symbolic interaction, both parties continually adjust their behavioral expectations and interaction patterns, thereby gradually deepening and solidifying interpersonal relationships. This mechanism also finds support in Nancy Eisenberg's perspective. This mechanism also finds support in Nancy Eisenberg's perspective. Eisenberg's series of studies repeatedly confirm that empathy is one of the most significant and consistent predictors of prosocial behavior. It serves not only as the direct emotional force driving helping behavior but also as the cornerstone for building high-quality interpersonal relationships. Individuals who can accurately perceive and respond to others' emotional needs are better equipped to establish mutually trusting and supportive peer relationships. This positive interpersonal environment, in turn, provides ongoing opportunities and reinforcement for individuals to practice and consolidate prosocial behaviors (Eisenberg et al., 1999). Therefore, the empathy → interpersonal relationships pathway revealed in this study essentially concretizes the core role of empathy emphasized by Eisenberg within a dynamic, socially interactive process.

In summary, basketball intervention, as a quintessential interactive ritual, constructs a collective activity space for junior high students that is rich in frequent interactions, close cooperation, and complex scenarios. In this setting, students effectively stimulate emotional resonance among one another through physical coordination and rhythmic synchronization, forming a strong emotional bond that enhances their capacity for empathy. As the interactive ritual continues, participants gradually develop a sense of membership tied to shared symbolic recognition, further solidifying and deepening their interpersonal bonds. This process not only enhances students' sense of collective belonging but also inspires their intrinsic motivation to engage in prosocial behaviors that align with societal expectations.

### 4.5 Recommendation

The core contribution of this study lies not in proving basketball as the sole effective intervention program, but in demonstrating how any team sport with similar social interaction potential—such as soccer, volleyball, or others—can be transformed into an effective tool for promoting prosocial development through a teaching framework emphasizing cooperation, respect, empathy, and ethical reflection. Future research and practice should focus more on constructing and validating this universal teaching model, rather than simply promoting a specific sport.

### 4.6 Limitations

One limitation of this study is that the conventional physical education classes conducted in the control group may not have fully matched the experimental group's basketball intervention in terms of the social interactivity, depth of cooperation, and emotional engagement of the activities. This difference in dosage may partially explain the variation in outcomes between groups. Future research should design more active and socially interactive control activities to better isolate the unique effects of specific movement instruction models.

Another limitation lies in the small sample size and single-school origin of this study, which restricts the generalizability of the findings. Furthermore, despite employing a pre-test–post-test design, the quasi-experimental design of cluster randomization remains weaker in terms of causal inference strength than a true randomized controlled trial. These are all factors that require careful consideration when interpreting the findings of this study.

## 5 Conclusions

Exercise intervention can directly promote empathy, interpersonal relationships, and prosocial behavior levels, but the basketball-based exercise intervention yields significantly greater effects than the conventional physical education class group. Exercise intervention can also indirectly enhance prosocial behavior among junior high students through the separate mediating effects of empathy and interpersonal relationships. Empathy and interpersonal relationships exhibit a chain-like mediating effect between exercise intervention and prosocial behavior.

## Data Availability

The raw data supporting the conclusions of this article will be made available by the authors, without undue reservation.
